# The Mediating Role of Human Mobility in Temporal-Lagged Relationships Between Risk Perception and COVID-19 Dynamics in Taiwan: Statistical Modeling for Comparing the Pre-Omicron and Omicron Eras

**DOI:** 10.2196/55183

**Published:** 2024-08-20

**Authors:** Min-Chien Chang, Tzai-Hung Wen

**Affiliations:** 1 Department of Geography, National Taiwan University, Taipei, Taiwan

**Keywords:** human mobility, risk perception, COVID-19, Omicron, Taiwan, pandemic, disease transmission, pandemic dynamics, global threats, infectious disease, behavioural health, public health, surveillance

## Abstract

**Background:**

The COVID-19 pandemic has profoundly impacted all aspects of human life for over 3 years. Understanding the evolution of public risk perception during these periods is crucial. Few studies explore the mechanisms for reducing disease transmission due to risk perception. Thus, we hypothesize that changes in human mobility play a mediating role between risk perception and the progression of the pandemic.

**Objective:**

The study aims to explore how various forms of human mobility, including essential, nonessential, and job-related behaviors, mediate the temporal relationships between risk perception and pandemic dynamics.

**Methods:**

We used distributed-lag linear structural equation models to compare the mediating impact of human mobility across different virus variant periods. These models examined the temporal dynamics and time-lagged effects among risk perception, changes in mobility, and virus transmission in Taiwan, focusing on two distinct periods: (1) April-August 2021 (pre-Omicron era) and (2) February-September 2022 (Omicron era).

**Results:**

In the pre-Omicron era, our findings showed that an increase in public risk perception correlated with significant reductions in COVID-19 cases across various types of mobility within specific time frames. Specifically, we observed a decrease of 5.59 (95% CI −4.35 to −6.83) COVID-19 cases per million individuals after 7 weeks in nonessential mobility, while essential mobility demonstrated a reduction of 10.73 (95% CI −9.6030 to −11.8615) cases after 8 weeks. Additionally, job-related mobility resulted in a decrease of 3.96 (95% CI −3.5039 to −4.4254) cases after 11 weeks. However, during the Omicron era, these effects notably diminished. A reduction of 0.85 (95% CI −1.0046 to −0.6953) cases through nonessential mobility after 10 weeks and a decrease of 0.69 (95% CI −0.7827 to −0.6054) cases through essential mobility after 12 weeks were observed.

**Conclusions:**

This study confirms that changes in mobility serve as a mediating factor between heightened risk perception and pandemic mitigation in both pre-Omicron and Omicron periods. This suggests that elevating risk perception is notably effective in impeding virus progression, especially when vaccines are unavailable or their coverage remains limited. Our findings provide significant value for health authorities in devising policies to address the global threats posed by emerging infectious diseases.

## Introduction

The COVID-19 pandemic has significantly impacted human life, with human mobility serving as a crucial factor in contagious disease transmission [1]. Throughout the pandemic, human mobility was shaped by both mandatory preventive measures and the evolving risk perceptions of the public. Different countries and cultures have responded to the pandemic with varying strategies and levels of effectiveness. For example, Japan initially implemented voluntary lockdowns and relied on its citizens’ compliance without enforcing strict legal penalties. Italy, severely hit in the early stages, imposed stringent lockdowns and travel restrictions to control the virus spread. Switzerland adopted a more flexible approach with phased lockdowns and later relied on public adherence to guidelines [2-5]. These varying responses highlight the importance of understanding context-specific public risk perceptions and mobility patterns.

Risk perception varied across different phases of the pandemic. Initially, when vaccines were scarce and unavailable, there was widespread fear of infection, leading to behaviors such as home confinement and avoiding gatherings [6]. As vaccination coverage increased and cases presented milder symptoms or were asymptomatic, public fear diminished and a strong desire to return to normal life emerged [7-9]. Understanding the evolution of public risk perception during the pandemic is critical for tailoring effective policy responses, implementing health education, and comprehending shifting behaviors, essential for long-term crisis management and adaptability [10].

Individuals’ compliance with preventive measures is influenced by their levels of fear of infection and their awareness of risks. Previous studies have confirmed the association between risk perception and human mobility. Risk perception is also correlated with various factors such as internet search queries, which reflect near real-time “individual fear of infection” [11,12], and the rate of vaccination and flu trends [13,14], all of which are useful for COVID-19 trend forecasts. Additionally, forecasting studies using statistical or machine learning methods, as well as surveillance studies using compartmental or agent-based models, have incorporated mobility as a crucial factor or covariate in predicting COVID-19 trends [11,12]. Those with a heightened risk perception during the pandemic are more likely to engage in preventive behaviors [6,15]. However, as the pandemic persists, individuals might experience a decline in motivation to adhere to safety measures, such as mask-wearing, maintaining social distance, and practicing proper hand hygiene. This decline may stem from an improved pandemic situation or an increase in psychological fatigue due to prolonged periods of isolation or avoidance of gatherings [16,17]. Diminishing adherence carries significant public health implications, potentially leading to a surge in COVID-19 cases and undermining containment efforts, particularly during the transition to the post-pandemic phase [18].

Recent studies underscore that mobility involving frequent close contact and enclosed settings, such as recreational activities and workplace interactions, pose higher risks of transmission [19-21]. In contrast, activities like grocery shopping and pharmacy visits usually entail briefer interactions, leading to reduced transmission risks [22]. Considering the time lag is crucial to understanding the significant impact of social distancing measures in flattening the epidemic curve, with their effects becoming evident 5‐15 weeks after implementation [23]. Recognizing the tangible effects of changes in human mobility takes time to manifest due to the characteristics of the disease, including its incubation period and the mode of transmission through respiratory droplets [24-26].

In summary, we have known that human mobility is intricately linked not only to the COVID-19 pandemic but also influenced by public risk perception evolving over time. However, there is limited understanding of the role played by changes in human mobility between risk perception and the course of the COVID-19 pandemic during different variant periods. To elucidate this mechanism, it is crucial to consider the temporal dynamics between risk perception, human mobility, and the progression of the pandemic.

**Figure 1.**
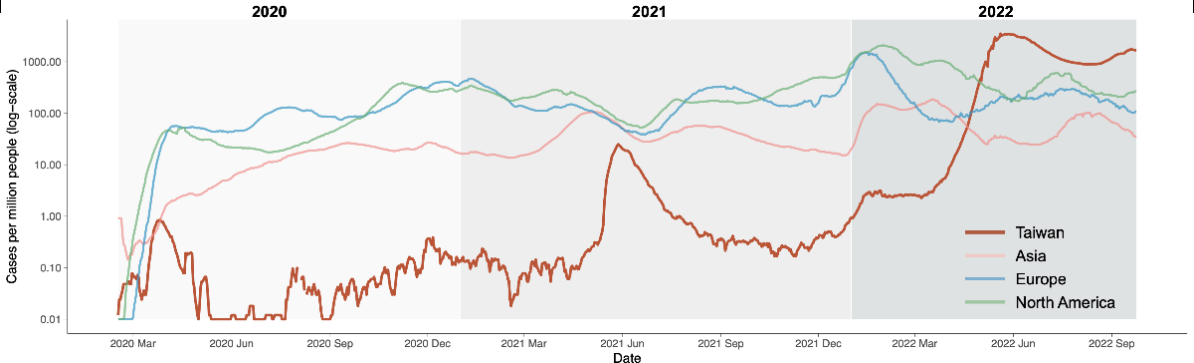
The distribution of COVID-19–confirmed cases per million people (log-scale) from February 2020 to September 2022 (Source: Johns Hopkins University Center for Systems Science and Engineering COVID-19 data).

In Taiwan, the COVID-19 pandemic unfolded in 2 distinct waves. As illustrated in Figure 1, these waves were contained within Taiwan, unaffected by external epidemic fluctuations, owing to effective border control measures. The pandemic remained relatively mild until 2021, when the emergence of the Alpha variant sparked the first significant outbreak, prompting fear and concern due to the absence of vaccines [27,28]. In contrast, the 2022 outbreak, driven by the Omicron variant, saw exponential growth. With a significant portion of the population having received 2 vaccine doses, the government gradually relaxed border restrictions and domestic containment measures [29,30]. This relaxation potentially led to a more complacent public risk perception, resulting in reduced compliance with public health recommendations. These distinct waves in Taiwan underscore the variations in public risk perception and its consequential impact on public behaviors, thus shaping the trajectory of the pandemic.

Our study aims to investigate the mediating role of human mobility in the relationship between public risk perception and the progression of the COVID-19 pandemic in Taiwan. We have two primary objectives and they are (1) to examine the time-lagged impact of evolving public risk perception on the pandemic, focusing on changes in various forms of human mobility, including essential, nonessential, and job-related behaviors, as mediators during the extended pandemic period and (2) to compare the impact of risk perception between the pre-Omicron and Omicron eras, analyzing how the distinct characteristics of these periods influence the relationship between risk perception, mobility, and virus transmission.

Our hypotheses are as follows.

**Figure 2.**
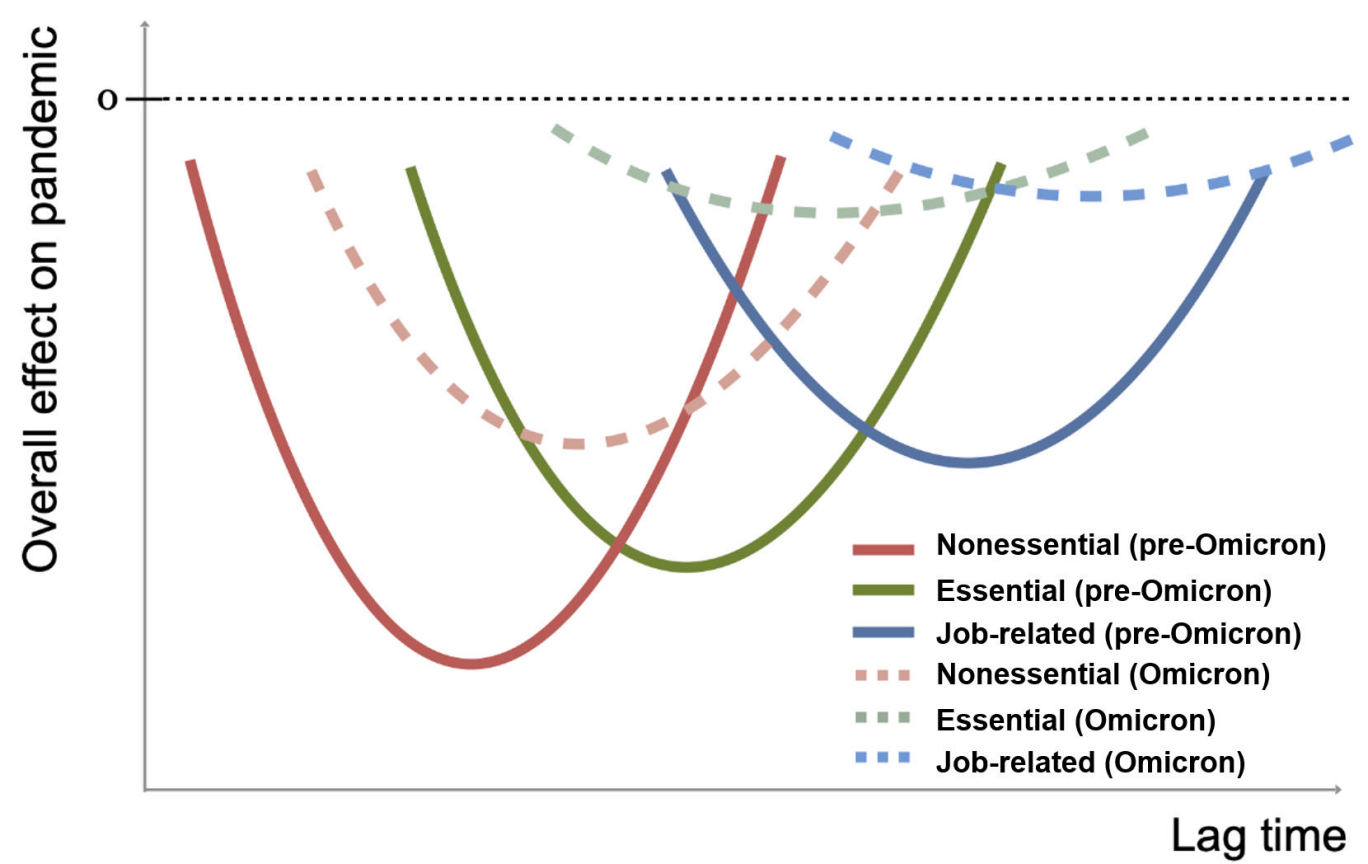
Hypothesized diagram of the overall effect of risk perception on the pandemic.

- H1: Heightened public risk perception may lead to subsequent reductions in virus transmission, potentially mediated by changes in mobility. We hypothesize that the effect of heightened public risk perception will follow a U-shaped pattern, becoming more pronounced initially and then diminishing as the epidemic situation improves, as depicted in Figure 2.
- H2: During the Omicron era, reduced public risk perception might lead to a delayed decline in confirmed cases due to the heightened transmissibility of the variant and decreased adherence to social distancing. We hypothesize that the time-lag effect in the Omicron era will occur later, represented by the dashed line in Figure 2.
- H3: In the pre-Omicron era, all forms of mobility could act as mediators, whereas in the Omicron era, essential and job-related mobility might have a diminished impact on the relationship between risk perception and virus transmission. We hypothesize that this reduction in impact could be due to the pressing need for normalcy in daily life and livelihood. Figure 2 illustrates this with relatively flat green and blue dotted lines.

## Methods

### Ethical Considerations

All data are from publicly available sources without any approval requirement.

### Study Design and Data Source

In early April 2021, Taiwan faced a small-scale outbreak of the epidemic when vaccination coverage was low. Public awareness of the virus’s threat was notably high. However, by late August 2021, the number of new daily confirmed cases had been successfully reduced to fewer than 10 people due to the implementation of the “zero-COVID” strategy. Subsequently, in late February 2022, over 70% of the Taiwanese population had received 2 doses of the COVID-19 vaccine. With this substantial vaccine coverage, the government began gradually relaxing pandemic regulations in September 2022. As such, this study focuses on 2 distinct periods—the “pre-Omicron era,” spanning from April 4, 2021, to August 29, 2021, and the “Omicron era,” covering the time frame from February 27, 2022, to September 18, 2022.

**Figure 3.**
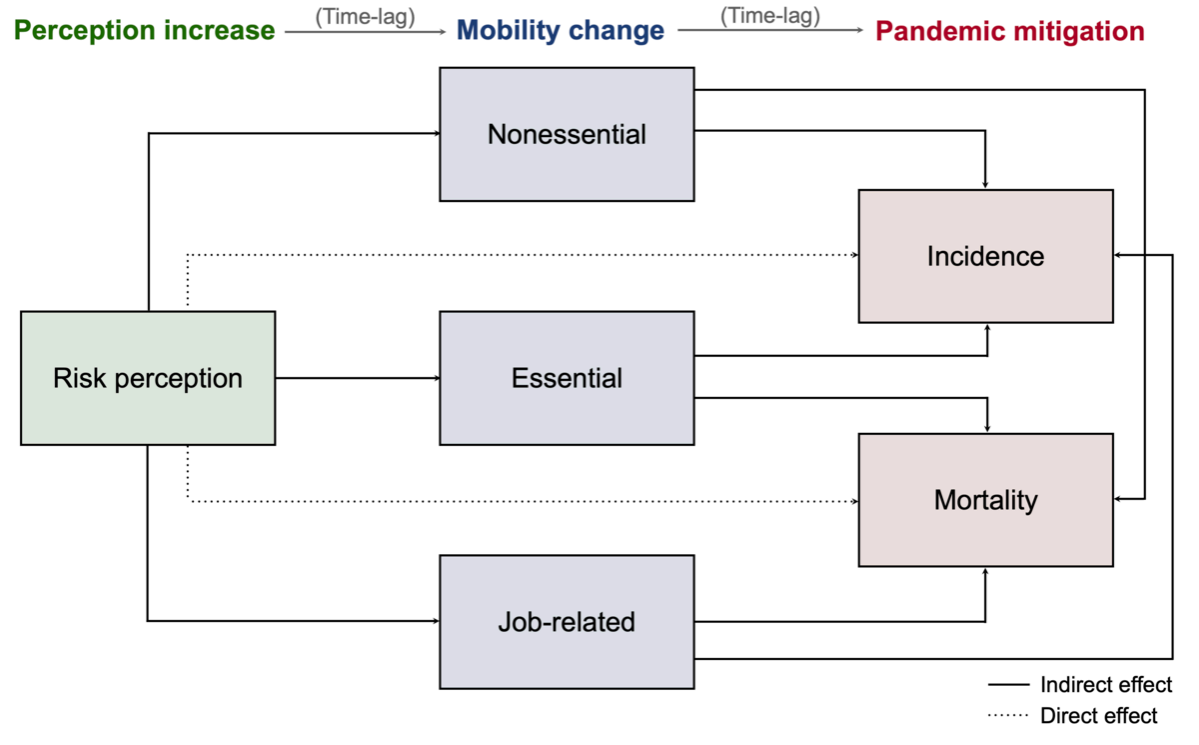
The directed acyclic graph of the model framework (considering domestic nonpharmaceutical intervention and vacation as control variables).

The analysis used a longitudinal design to evaluate the mediating impact of human mobility between public risk perception and the progression of the COVID-19 pandemic, considering the time lag for each path represented in Figure 3.

This study examines the role of 3 types of mobility changes—essential, nonessential, and job-related behaviors—as mediating factors in understanding their influence between risk perception and disease transmission. Nonessential mobility typically pertains to leisure activities, providing individuals with stress relief and enjoyment. Such activities are discretionary and can be engaged in or canceled based on individual preferences. For our study, Google’s Community Mobility Reports’ “Retail and recreation” category, covering places like restaurants, cafes, shopping centers, and more represents nonessential mobility.

On the other hand, essential mobility involves acquiring necessary goods for daily life, such as food and medicines. These activities are critical for sustaining daily life and are indispensable. We considered Google’s “Grocery stores and pharmacies” category in the Community Mobility Reports to represent essential mobility, including supermarkets, drug stores, and other similar locations. Furthermore, job-related activities are tied to livelihoods and occupations. Shutdowns affecting these activities may have adverse financial implications, making it challenging for individuals to maintain their regular living standards. In our analysis, “Workplaces” from Google’s Community Mobility Reports represent economic or employment-related activities, considered as job-related mobility.

Our analysis compiles data on a weekly basis. Public risk perception data, measured by individuals’ information-seeking behavior, are sourced from Google Trends [31]. Specifically, the data extracted from Google Trends include the time series of particular search terms and topics related to COVID-19, such as “COVID-19 symptoms,” “COVID-19 prevention,” and “COVID-19 vaccine,” which collectively represent the public’s risk perception. COVID-19 case and mortality data are obtained from Johns Hopkins University [32]. Mobility change variables are calculated by averaging daily data retrieved from Google Community Mobility Reports. To address potential confounding factors, the OxCGRT reported 19 daily indices, enabling the calculation of weekly domestic nonpharmaceutical interventions (NPIs) by summing daily stringency index scores per week [33]. Our analysis integrates daily country-level data on COVID-19 domestic containment, including 7 indicators—school closures, workplace restrictions, event cancellations, limitations on gathering size, public transport closures, stay-at-home orders, and constraints on internal movement. The variable depicting Taiwan’s holidays is derived by aggregating the total number of holidays for each week.

### Statistical Analysis

We used the distributed-lag linear structural equation models (DLSEMs), which rely on Markovian structural causal models (MSCMs), for examining the mediating impact of human mobility between public risk perception and the pandemic progression. DLSEMs provide a mathematical framework for causal inference and offer a way to examine the temporal dynamics and lagged effects among variables [34] and offer an advantage by allowing the inclusion of lagged terms of independent variables in regression equations, effectively capturing the temporal dynamics and potential time delays in the relationship between variables, and providing a more comprehensive understanding of the complex interplay over time [35,36].

DLSEM is a statistical method that combines the concepts of structural equation modeling (SEM) and distributed-lag models (DLMs). SEM can simultaneously analyze multiple dependent and independent variables and consider complex relationships between latent variables. It can construct and test multiple causal paths, aiding in the understanding of causal relationships between variables. However, SEM is not effective in handling time-delayed relationships among variables [37].

On the other hand, DLMs are suitable for handling time series data, taking into account the lag effects of variables over different periods. It captures the dynamic influence of independent variables on dependent variables, making it suitable for studying relationships that change over time. However, DLMs typically can only handle the effect of a single independent variable on a dependent variable, unlike SEM, which can handle multiple variables simultaneously [38,39].

We chose DLSEMs because they combine the advantages of SEM and DLMs. DLSEMs can include lagged terms of independent variables in the regression equations, effectively capturing the temporal dynamics and potential time delay relationships between variables. This provides a more comprehensive understanding of complex interactions over time, which is crucial for understanding the dynamic relationships between risk perception, mobility, and the development of an epidemic, especially in a constantly evolving situation like a pandemic. Technical details of DLSEMs are described as follows.

In this study, DLSEMs formulate an MSCM on the set of variables as joint probability distributions, as follows:



(1)
$$p\left(V_{1},V_{2},V_{3},V_{4},V_{5}\right)=\prod_{j=1}^{5}p\left(V_{j}|\Pi_{j}\right)$$

(2)
$$p\left(V_{1},V_{2},V_{3},V_{4},V_{6}\right)=\prod_{j=1}^{6}p\left(V_{j}|\Pi_{j}\right)$$

Where for *j*>1, $V_{j}$ is independent of variables in ${\{V_{1},\ldots ,V}_{j-1}\}|\Pi_{j}$ given variables in $\Pi_{j}$. $V_{1}$ is risk perception, $V_{2}$ is nonessential mobility, $V_{3}$ is essential mobility, $V_{4}$ is job-related mobility, $V_{5}$ is COVID-19 confirmed cases per million people, and $V_{6}$ is COVID-19 deaths per million people.

In a DLSEM, each factor of the joint probability distributions in equations 1 and 2 is a distributed-lag linear regression, where $v_{j,t}$ is the response variable *j* at time *t* and variables in $\Pi_{j}$ are the covariates. Thus, a distributed-lag linear regression can be formulated as follows:



(3)
$$v_{j,t}=\alpha_{j}+\sum_{i:\forall V\in \Pi }\sum_{l=0}^{L_{i}}\beta_{j|i,l\;}v_{i,t-l\;}+\;\epsilon_{t}\;\;\;\;\;\;\;\epsilon ∼N\left(0,\sigma^{2}\right)$$

The set of coefficients $\beta_{j|i}=\beta_{j|i,0},\beta_{j|i,1,}\ldots ,\beta_{j|i,L_{i}}$ is denoted as the lag shape of $v_{j,t}$ and represents its influence on $v_{j,t-l}$ at different time lag (*t - l*).

In each regression model, we applied an end point–constrained quadratic lag shape to all covariates not belonging to the context level, with the following constraints—for estimating the effect of risk perception on human mobility change, we assume that a maximum gestation lag of 1 week, a minimum lag width of 1 week, and a maximum lead-lag of 5 weeks. For estimating the effect of mobility on the development of the COVID-19 pandemic, we set a maximum gestation lag of 3 weeks, a minimum lag width of 1 week, and a maximum lead-lag of 15 weeks based on existing studies [23]. Based on our hypothesis, we expect that the impact of risk perception starts off relatively small, gradually increases to a peak, and then diminishes to 0 after a certain number of time lags. To align with this hypothesis and ensure that our model reflects our prior knowledge of the phenomenon, we used the end point–constrained quadratic lag shape in our analysis. All statistical analyses were conducted with R (version 4.2.3; R Core Team). The “dlsem” package (version 2.4.6) was used for DLSEM.

## Results

### Descriptive Epidemiology

**Figure 4.**
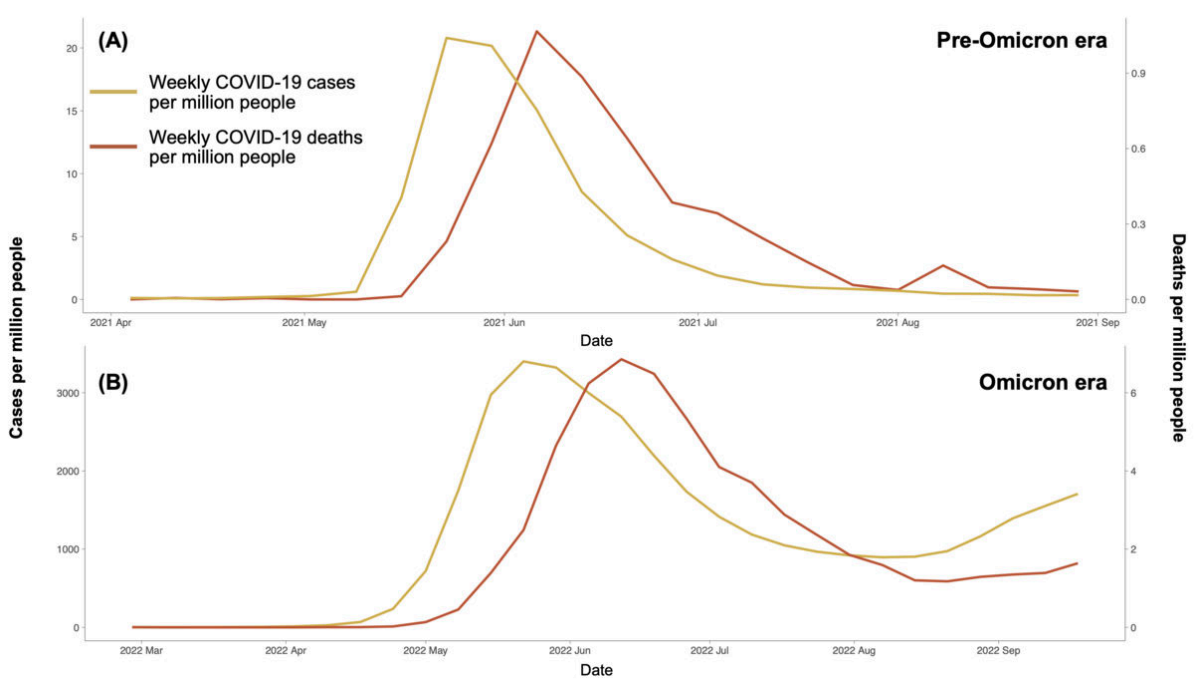
Weekly COVID-19 confirmed cases and deaths per million people in Taiwan (A) during COVID-19 pre-Omicron era and (B) Omicron era.

**Figure 5.**
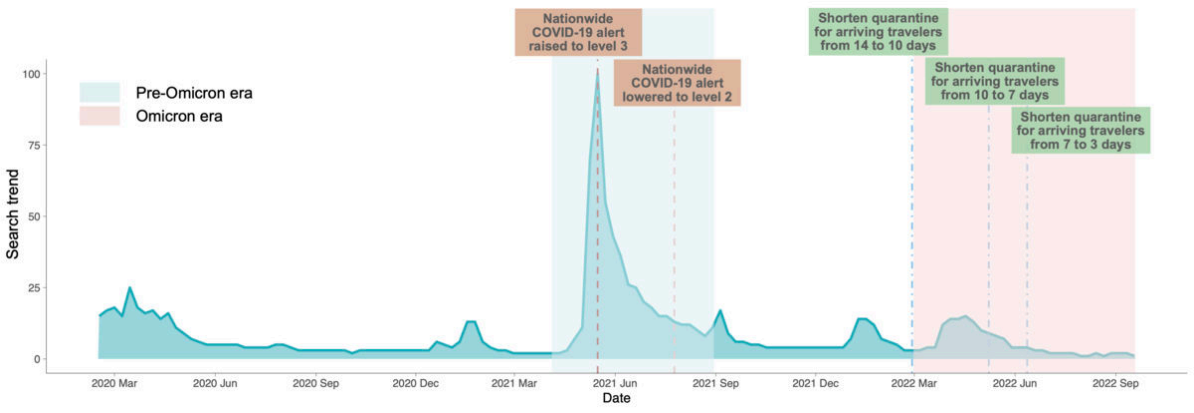
Weekly search trend in Taiwan (topic search: COVID-19) from February 2020 to September 2022.

The number of confirmed cases and deaths during the 2021 outbreak in Taiwan was 21 and 1 per million people, respectively. This was markedly lower compared to 3400 confirmed cases and 7 deaths per million people, respectively, during the 2022 Omicron outbreak, as illustrated in Figure 4. Notably, during the initial COVID-19 outbreak in Taiwan, there was a shortage of vaccines and essential supplies, prompting the government to implement strict NPI measures, particularly national alert level 3. This led to significant fear, anxiety, and a surge in information-seeking behavior among the public, as illustrated in Figure 5. In contrast, during the Omicron era, 70% of the population had received 2 vaccine doses, and the government gradually relaxed preventive measures, resulting in a lower risk perception among the population compared to the initial outbreak. These differences highlight the evolving public response and governmental measures across different phases of the pandemic, emphasizing the importance of understanding risk perception and mobility patterns.

**Figure 6.**
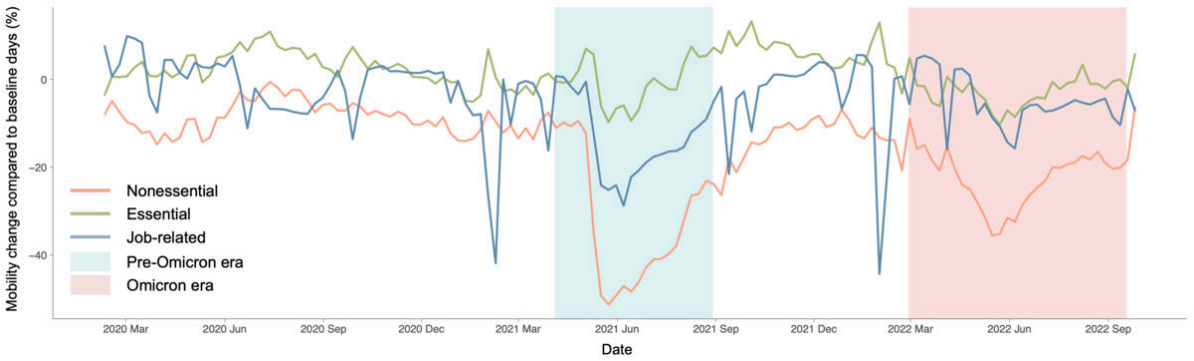
Time series of human mobility change for Taiwan from February 2020 to September 2022.

**Figure 7.**
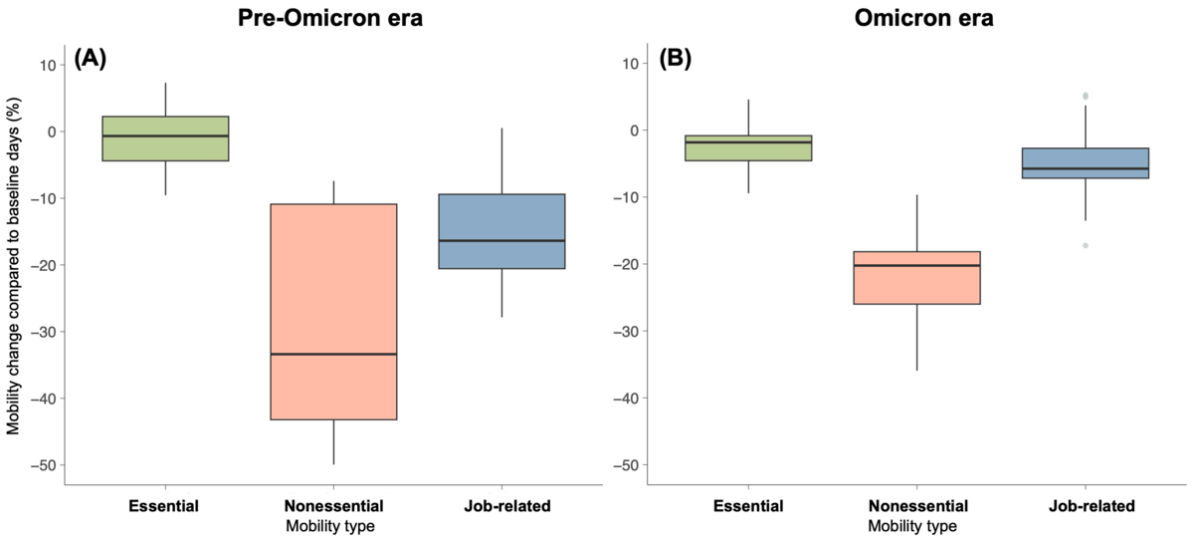
Human mobility change for Taiwan during (A) the pre-Omicron and (B) Omicron eras.

There was a significant decrease in human mobility in May 2021 in Taiwan, as shown in Figure 6. This pattern was particularly pronounced during the first outbreak in 2021, caused by the Alpha variant. In the pre-Omicron era, nonessential mobility displays the most significant variation, followed by job-related mobility, illustrated in Figure 7. Essential mobility shows minimal differences compared to the pre-Omicron era. In the Omicron era, the variation in nonessential mobility reduced compared to the pre-Omicron era, yet it still maintained a 20% decrease relative to the pre-Omicron era. Job-related mobility experiences a 10% decrease relative to the pre-Omicron era. These mobility trends suggest different public compliance levels and changes in daily activities, reflecting varying responses to government policies and perceived risk during different pandemic phases.

### Model Estimation Results

**Figure 8.**
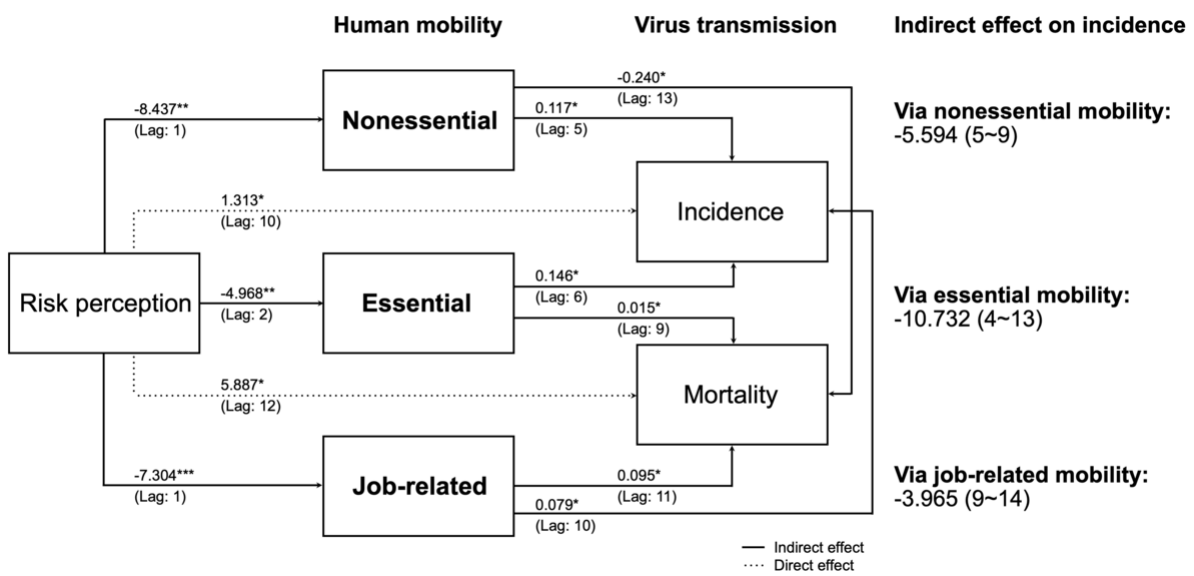
The pathway of the distributed-lag linear structural equation model with estimated causal effects and cumulative effects through each mediating factor (on the right) during the pre-Omicron era. The numbers on the arrows represent the coefficients of the peak effect, with the numbers in parentheses indicating the time delay at which the maximum effect occurs. (Domestic nonpharmaceutical intervention and vacation are considered as control variables). *P<.05; **P<.01; ***<.001.

**Figure 9.**
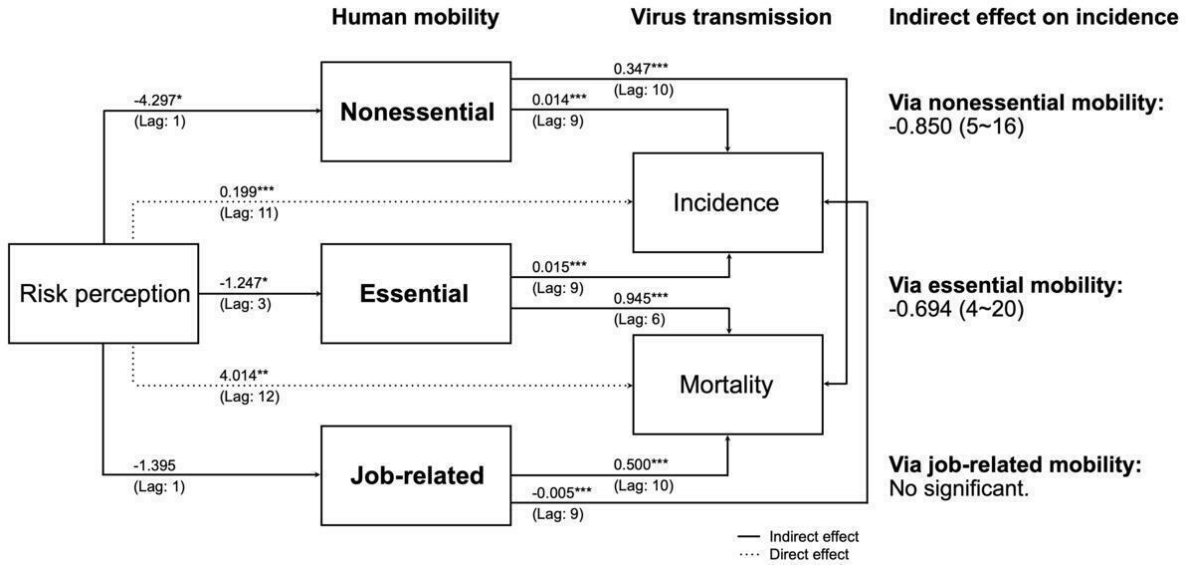
The pathway of the distributed-lag linear structural equation model with estimated causal effects and cumulative effects through each mediating factor (on the right) during the Omicron era. The numbers on the arrows represent the coefficients of the peak effect, with the numbers in parentheses indicating the time delay at which the maximum effect occurs. (Domestic nonpharmaceutical intervention and vacation are considered as control variables). *P<.05; **P<.01; ***<.001.

Figure 8 and Figure 9 illustrate the complete path of the structural equation model for the pre-Omicron and Omicron eras in Taiwan, respectively. During the pre-Omicron era, the increased risk perception associated with cases was significantly mediated by all types of human mobility. In the Omicron era, the increased risk perception associated with cases was significantly mediated by non-essential and essential mobility. This means that public fear of infection led to reduced movement, which in turn helped lower the number of new cases. This effect was more pronounced before the Omicron variant became widespread.

In the pre-Omicron era, following a 1- to 2-week period of heightened public risk perception, there was a peak decrease of 8.44 (95% CI −5.82 to −11.05; *P*=.020) in nonessential mobility, 4.97% (95% CI −3.74 to −6.20; *P*=.001) in essential mobility and 7.30% (95% CI −5.98 to −8.63; *P*<.001) in job-related mobility. After the decline in all types of mobility, reducing nonessential mobility led to a decrease of 0.12 (95% CI 0.11-0.12; *P*=.030) confirmed cases per million people within 5 weeks, essential mobility contributed to a reduction of 0.15 (95% CI 0.14-0.15; *P*=.020) within 6 weeks, and job-related mobility resulted in a decrease of 0.08 (95% CI 0.077- 0.08; *P*=.010) within 10 weeks.

In the Omicron era, after a 1- to 3-week period of heightened public risk perception, there was a peak decrease of 4.297 (95% CI −6.01 to −2.58; *P*=.020) in nonessential mobility and 1.25 (95% CI −1.84 to −0.66; *P*=.047) in essential mobility. After the decline in these two types of mobility, reducing nonessential mobility led to a decrease of 0.014 (95% CI 0.013-0.015; *P*<.001) confirmed cases per million people within 9 weeks, and essential mobility contributed to a reduction of 0.015 (95% CI 0.014-0.16; *P*<.001) within 9 weeks. These results demonstrate the delayed effects of risk perception on mobility and, consequently, on virus transmission. The changes in public behavior influenced by perceived risk had measurable impacts on the number of COVID-19 cases.

### The Overall Effect of Risk Perception on the COVID-19 Pandemic

**Figure 10.**
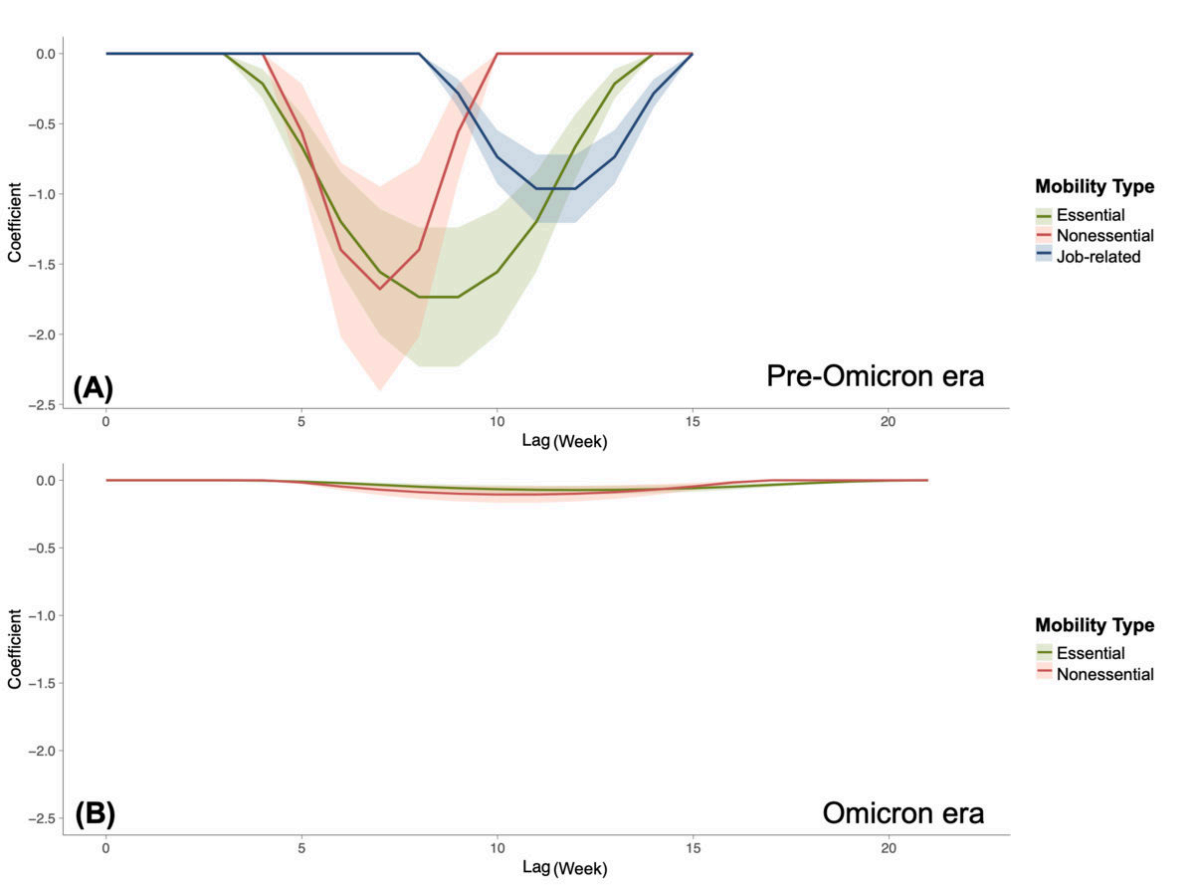
The mediating effect of human mobility between risk perception and COVID-19 incidence (A) during pre-Omicron era and (B) Omicron era in Taiwan (95% CIs are shown in a lighter color).

Figure 10 demonstrates that during the pre-Omicron era, risk perception generally led to a decrease in confirmed cases after a lag of 4 to 9 weeks. Similarly, during the Omicron era in Taiwan, risk perception generally led to a decrease in confirmed cases after a lag of 4 to 11 weeks.

All types of human mobility act as mediating factors in reducing the number of confirmed cases. Risk perception has a direct positive effect on the development of the pandemic, indicating that other factors may contribute to the escalation of the pandemic. The most significant effect on the number of confirmed cases is observed approximately 7 to 11 weeks after an increase in risk perception.

### Effect of Different Human Mobility as Mediators

We further investigate the effect and time lags related to different types of human mobility. In a pre-Omicron era in Taiwan, the overall effect of risk perception on confirmed cases was influenced by all types of mobility, with nonessential and essential mobility having an earlier onset on confirmed cases, as shown in Figure 10.

In the pre-Omicron era, a 1-unit increase in public risk perception is associated with a reduction of 5.59 (95% CI −4.35 to −6.83) COVID-19 cases per million people through nonessential mobility after 7 weeks. Essential mobility exhibits a reduction of 10.73 (95% CI −9.6030 to −11.8615) COVID-19 cases per million people after 8 weeks, while job-related mobility results in a decrease of 3.96 (95% CI −3.5039 to −4.4254) COVID-19 cases per million people after 11 weeks. The cumulative effect of mediating factors shown in Table 1 reveals that essential mobility has a substantial impact.

In the Omicron era in Taiwan, the overall effect of risk perception on confirmed cases was influenced by nonessential and essential mobility, as shown in Figure 10.

During the Omicron era, a 1-unit increase in public risk perception leads to an anticipated reduction of 0.85 (95% CI −1.0046 to −0.6953) COVID-19 cases per million people through recreational mobility after 10 weeks. Essential mobility shows a reduction of 0.69 (95% CI −0.7827 to −0.6054) COVID-19 cases per million people after 12 weeks. The cumulative effect of mediating factors in the Omicron era is much smaller than in the pre-Omicron era, as we saw in Figure 10.

These findings highlight how different types of mobility play varying roles in mediating the impact of risk perception on COVID-19 transmission. The more pronounced effects in the pre-Omicron era reflect the stricter public response to the initial stages of the pandemic compared to the more relaxed attitudes during the Omicron era.

**Table 1.** Summary of the overall causal effect associated to each path, linking risk perception to human mobility change, and then connecting confirmed cases in Taiwan.

Pathway		Peak temporal-lagged effect(week)	Estimate (SE)		95% CI	
**Pre-Omicron**						
	Risk→nonessential→cases	7	–5.5940 (0.6328)		−6.8342 to −4.3538	
	Risk→essential→cases	8	–10.7323 (0.5762)		−11.8615 to −9.6030	
	Risk→job-related→cases	11	–3.9646 (0.2351)		−4.4254 to −3.5039	
	Risk→cases	10	8.6680 (0.0549)		8.5605 to 8.7755	
	Overall causal effect on cases	7	–11.6229 (1.2867)		−14.1448 to −9.1010	
**Omicron**						
	Risk→nonessential→cases	10	–0.8499 (0.0789)		−1.0046 to −0.6953	
	Risk→essential→cases	12	–0.6941 (0.0452)		−0.7827 to −0.6054	
	Risk→cases	11	0.3538 (0.0423)		0.2709 to 0.4366	
	Overall causal effect on cases	10	−1.1902 (0.1491)		−1.4824 to −0.8981	

## Discussion

### Summary of Findings

The findings of our study provide valuable insights into how shifts in public risk perception impact the COVID-19 pandemic through human mobility across distinct pandemic phases. First, our analysis reveals a nonlinear relationship between human mobility and the association of public risk perception with COVID-19 transmission. This evidence indicates that risk perception can shape the dynamics of the COVID-19 pandemic through alterations in human mobility, displaying a U-shaped pattern over time. Second, the influence of risk perception on virus transmission through human mobility differs across various stages. Its impact persists longer but exhibits a lesser effect during the Omicron era compared to the pre-Omicron era. Finally, in the pre-Omicron era, essential, nonessential, and job-related mobility collectively mediate the link between public risk perception and COVID-19 transmission. However, in the Omicron era, job-related mobility does not significantly mediate the relationship between risk perception and disease transmission. A common aspect observed across both pandemic eras is the earlier discernible impact of risk perception on COVID-19 transmission for essential and nonessential mobility compared to job-related mobility.

### Mechanism of Pandemic Mitigation Measures

The findings suggest that an upsurge in public risk perception corresponds to a decrease in human mobility, corroborating existing literature [6,15]. Similarly, a decline in human mobility contributes to future pandemic mitigation, aligning with established research [20,21,30]. Synthesizing these relationships from prior studies, our research indicates that heightened public risk perception leads to pandemic mitigation through reduced human mobility [16]. Our model delineates a U-shaped pattern in the relationship among these variables, consistent with findings in existing studies. This pattern implies an initial surge in public fear and concern at the pandemic’s onset, with gradual changes in mobility affecting pandemic mitigation. As the epidemic situation improves, public concerns diminish, and the impact of behavioral changes on disease mitigation lessens progressively [40,41]. Overlooking the time-varying aspect of this mechanism might underestimate pandemic mitigation effectiveness. Our research addresses this gap by using a time-varying model, offering a nuanced perspective on the large-scale impact of public mobility on the relationship between risk perception and epidemic status. This approach reveals a dynamic nature of epidemic mitigation not extensively discussed in previous studies.

### The Time Lag Effect of Risk Perception on Virus Transmission

Our findings confirm that shifts in mobility prompted by risk perception contribute to delayed pandemic mitigation during the Omicron era, in line with our initial hypothesis. The time lag in our study denotes the duration necessary for the effects of heightened public risk perception on pandemic control to manifest. During the Omicron era, we observed this duration ranged from 4 to 20 weeks after the elevation of risk perception, extending 6 weeks beyond the pre-Omicron era (4 to 14 weeks). Notably, previous studies conducted in Europe and the United States suggest that a decline in human mobility can initiate a slowdown in COVID-19 transmission within 2‐7 weeks [25,26,42]. The estimated onset times of these impacts within our models align with this range. However, during the Omicron era, the duration is notably longer, and the effect is markedly reduced. This phenomenon may be attributed to pandemic fatigue related to social distancing measures among the population, compounded by a substantial surge in confirmed cases in 2022, thereby prolonging the pandemic’s abatement period [16]. Furthermore, by reaching a 70% vaccination coverage rate, Taiwan effectively curtailed severe cases, subsequently leading to diminished public risk perception compared to the initial 2021 outbreak [43]. Previous studies have primarily focused on the 2020‐2021 pandemic [25,26,41,42]. Building upon this foundation, our research analyzes the delayed impact of risk perception on virus transmission across different pandemic phases. In comparison to prior studies, our research confirms that during the Omicron period, the duration of this effect is longer, indicating varying influences of pandemic fatigue and vaccination coverage on public behavior and epidemic outcomes across different phases of the pandemic. This adds a new dimension to understanding the dynamics of infectious diseases over time.

### Mediation by Different Human Mobility Types of the Association Between Risk Perception and COVID-19 Transmission

In the pre-Omicron era, before achieving high vaccine coverage rates, all categories of mobility were identified as significant mediators. Our findings may suggest that interactions within nonessential activities, such as recreational pursuits, and job-related activities, like daily commutes, could potentially serve as high-risk transmission routes due to close contact and enclosed environments. Previous studies consistently highlight that changes in these job-related and nonessential mobilities are positively correlated with future virus transmission and pandemic severity in many countries [21,22,44-47].

Conversely, essential activities like grocery shopping and pharmacy visits typically involve shorter interaction durations, resulting in reduced transmission risks [20,48]. Recent studies suggest that essential mobility did not significantly impact virus transmission in the United States throughout 2020‐2021 [24,47,49]. However, our study observed a significant impact of essential mobility on virus transmission, suggesting a nuanced difference in the Taiwanese context. It indicates that essential mobility acts as a mediator between risk perception and virus transmission throughout 2021‐2022, albeit with a weaker mediating effect in 2022 during the Omicron era compared to the effect observed in 2021. In the pre-Omicron era, this could be attributed to people becoming more cautious in their shopping routines, emphasizing hygiene practices, and adopting contactless shopping modes. Widespread use of face masks and hand sanitizers, and adherence to social distancing measures became standard practices in grocery stores, pharmacies, and other essential shopping locations [50,51]. The situation in 2022 during the Omicron era can be attributed to the effectiveness of a high vaccination rate among the population, leading to the normalization of essential mobility. These adaptive shopping behaviors became pivotal in the public’s overall risk management strategy against a highly transmissible virus.

Taiwan’s epidemic intervention strategy played a crucial role in mitigating the impact of different types of activities. In comparison to many countries, Taiwan’s proactive and stringent public health measures, such as early border controls, comprehensive contact tracing, and effective quarantine methods, have contributed to a more effective containment of virus spread [52]. These measures, coupled with high levels of compliance and trust in government directives among the public, further influenced risk perception and activity patterns. Thus, the stringency and timely implementation of these policies may have contributed to differences in the mediating effects of mobility types across different stages of the pandemic.

When comparing the peak times and scales across different types of mobility, we observed that essential and nonessential mobility exhibited similar peak times and scales during the pre-Omicron era. However, job-related mobility displayed a smaller scale and a delayed peak time. This difference could be attributed to the significant costs associated with workplace closure measures, despite their effectiveness [53]. Additionally, commuting behaviors remained relatively unchanged before and after the pandemic [54]. Our findings underscore the importance of considering various types of mobility in relation to the association between risk perception and future pandemic outcomes when devising targeted interventions and preventive strategies.

### Policy Implications

Our findings carry significant policy implications for health authorities. Human mobility, influenced by risk perception, significantly shapes future pandemics in a dynamic process. Health authorities should consider that the impact of public health measures on the pandemic is not immediate. Our study identifies a relevant time frame illustrating how mobility changes impact the occurrence of the pandemic. This insight provides a valuable reference for determining the optimal timing to ease restrictive policies. Prior to the widespread availability of effective vaccines, it is imperative to prioritize the accurate dissemination of information concerning the severity of the pandemic, its transmission pathways, and essential facts. Public concerns and apprehensions are notably linked to nonessential and essential mobility, influencing pandemic mitigation more than job-related activities. Thus, our findings could provide insights for implementing preventive measures against future emerging infectious diseases.

As the Omicron period witnesses increased vaccine accessibility and relaxed government mobility restrictions, sustaining efforts in enhancing public health education remains pivotal. Sensitizing individuals to risk perception encourages the voluntary adoption of preventive behaviors, ensuring a sustainable strategy for long-term pandemic management. Additionally, understanding the delayed effects of public risk perception on virus transmission through changes in mobility can help health authorities better allocate resources and adjust policies timely. This dynamic understanding can lead to more effective planning of medical resource distribution, such as vaccines and health care capacity.

### Limitations

This study has several limitations. First, using Google Mobility data as a proxy for changes in human mobility might underestimate the actual reasons behind trips. As the specific purposes of these movements remain undisclosed, our inferences are solely drawn from the locations people visited. These data primarily reflect population levels at these places rather than capturing individual behaviors. Second, all the data used in our study are aggregated, lacking the incorporation of individual lifestyle habits, such as mask-wearing, which could significantly impact the virus spread. Finally, the absence of individual-level data hindered the integration of individual behaviors into our model. While our model shows a reduction in the mediating effect during the Omicron era, it does not elucidate whether public behavioral changes are predominantly driven by individual willingness or the relaxation of government policies. Future research endeavors could integrate diverse survey methodologies to conduct a more comprehensive investigation into public risk perception and preventive behaviors, leveraging both macro-level and individual-level data. These limitations highlight the need for further research to address these challenges and obtain more appropriate measurements of risk perception and behavioral changes during pandemics.

### Conclusions

This study uncovers significant insights into the interplay between public risk perception, human mobility, and COVID-19 transmission across distinct pandemic phases. We found a nonlinear, U-shaped relationship between mobility and risk perception, with the impact of risk perception on virus transmission varying between the pre-Omicron and Omicron eras. In the pre-Omicron phase, all types of mobility mediated the association between risk perception and COVID-19 transmission, while in the Omicron era, job-related mobility showed no influence. These findings suggest that health authorities can optimize the timing and nature of public health interventions by understanding the time-lagged effects of risk perception on mobility. Our findings highlight the importance of maintaining robust public health education efforts to sustain voluntary preventive behaviors. Future research may further incorporate individual-level data to explore the nuanced impacts of specific behaviors on virus transmission, enhancing the effectiveness of pandemic responses and better preparing for future public health crises.

## Abbreviations

DLM: distributed-lag model
DLSEM: distributed-lag linear structural equation model
MSCM: Markovian structural causal model
NPI: nonpharmaceutical intervention
SEM: structural equation modeling
